# A statistical approach for segregating cognitive task stages from multivariate fMRI BOLD time series

**DOI:** 10.3389/fnhum.2015.00537

**Published:** 2015-10-07

**Authors:** Charmaine Demanuele, Florian Bähner, Michael M. Plichta, Peter Kirsch, Heike Tost, Andreas Meyer-Lindenberg, Daniel Durstewitz

**Affiliations:** ^1^Department of Theoretical Neuroscience, Bernstein Center for Computational Neuroscience, Central Institute of Mental Health, Medical Faculty Mannheim, Heidelberg UniversityMannheim, Germany; ^2^Department of Psychiatry and Psychotherapy, Central Institute of Mental Health, Medical Faculty Mannheim, Heidelberg UniversityMannheim, Germany; ^3^Athinoula A. Martinos Center for Biomedical Imaging, Massachusetts General Hospital, Harvard Medical SchoolBoston, MA, USA; ^4^Department of Clinical Psychology, Central Institute of Mental Health, Medical Faculty Mannheim, Heidelberg UniversityMannheim, Germany

**Keywords:** multivariate pattern analysis, working memory, decision making, prefrontal cortex, Hidden Markov Models, discriminant analysis, classifiers, machine learning

## Abstract

Multivariate pattern analysis can reveal new information from neuroimaging data to illuminate human cognition and its disturbances. Here, we develop a methodological approach, based on multivariate statistical/machine learning and time series analysis, to discern cognitive processing stages from functional magnetic resonance imaging (fMRI) blood oxygenation level dependent (BOLD) time series. We apply this method to data recorded from a group of healthy adults whilst performing a virtual reality version of the delayed win-shift radial arm maze (RAM) task. This task has been frequently used to study working memory and decision making in rodents. Using linear classifiers and multivariate test statistics in conjunction with time series bootstraps, we show that different cognitive stages of the task, as defined by the experimenter, namely, the *encoding/retrieval*, *choice, reward* and *delay* stages, can be statistically discriminated from the BOLD time series in brain areas relevant for decision making and working memory. Discrimination of these task stages was significantly reduced during poor behavioral performance in dorsolateral prefrontal cortex (DLPFC), but not in the primary visual cortex (V1). Experimenter-defined dissection of time series into class labels based on task structure was confirmed by an unsupervised, bottom-up approach based on Hidden Markov Models. Furthermore, we show that different groupings of recorded time points into cognitive event classes can be used to test hypotheses about the specific cognitive role of a given brain region during task execution. We found that whilst the DLPFC strongly differentiated between task stages associated with different memory loads, but not between different visual-spatial aspects, the reverse was true for V1. Our methodology illustrates how different aspects of cognitive information processing during one and the same task can be separated and attributed to specific brain regions based on information contained in multivariate patterns of voxel activity.

## Introduction

Functional magnetic resonance imaging (fMRI) has provided neuroscience with an invaluable tool for the investigation of cognitive functions, such as working memory and decision making (D’Esposito et al., 2000; Owen et al., 2005; D’Esposito, 2007; O’Doherty et al., 2007). Traditional experiments and fMRI analyses approaches have helped to attribute defined cognitive functions to specific brain regions and networks (Logothetis, 2008), and to identify impairments in these networks in various neuropsychiatric disorders such as schizophrenia (Meyer-Lindenberg et al., 2005; Esslinger et al., 2009; Gur and Gur, 2010; Deserno et al., 2012) and depression (Sheline et al., 2010; Forbes, 2011; Wang et al., 2012; Jaworska et al., 2014), yielding novel endophenotypes for these disorders (Meyer-Lindenberg and Weinberger, 2006; Meyer-Lindenberg, 2010). This could ultimately help target treatment and improve cognition and functional outcome of patients.

The most popular strategy for extracting such information from fMRI data is centered on the subtraction approach, where one isolates a task process of interest (such as, working memory) and designs a control task, which is identical to the target task except for the process of interest. By definition, this approach requires a control task to be implemented for every process of interest, and control trials have to be incorporated in sequence with target trials in the experimental setup. Typically, such task design provides information on whether a particular brain region is significantly more activated during the process of interest in comparison to control (Friston, 2007).

However, to gain deeper insight into cognitive processing, it is important to assess information processing in these regions throughout the course of the task. Multivariate methods provide a possible way to achieve this, by discerning more finely grained aspects of information processing from *patterns* of voxel activation that form the multivariate fMRI blood oxygenation level dependent (BOLD) time series (Haynes and Rees, 2006; Kriegeskorte et al., 2006; Norman et al., 2006; Haynes et al., 2007). These methods can illuminate different stages of cognitive processing and thereby enhance our understanding of the computations occurring in specific regions.

In this work, we suggest a novel conceptual strategy, based on multivariate classifiers and time-series bootstraps approaches, and comprising both supervised methods where time on task is split into experimenter-defined task stages, and an unsupervised, bottom-up (data-driven) approach based on Hidden Markov Models (Rabiner, 1989; Bishop, 2007) that is completely data driven. This approach may eliminate the need of a control task in cases where the main aim is not that of explicitly contrasting different task conditions for singling out specific brain areas. Using these methods, any process of interest can be isolated and attributed to a specific brain region by grouping time bins according to functional hypotheses, training the classifier, and testing significance by means of non-parametric time-series bootstraps (Davison and Hinkley, 1997).

We evaluate and validate this novel approach on a complex delayed win-shift radial arm maze (RAM) task in a group of healthy adults. In rodents, the RAM task has often been employed to study decision making and multiple-item working memory in an ecologically valid context (Olton and Samuelson, 1976; Cook et al., 1985; Brown and Giumetti, 2006; Lapish et al., 2008, 2015). This task involves navigating through a radial maze marked by various landmarks of different visual saliency, and memorizing the location of the arms to find hidden rewards. It implies many different motor, visual, memory, and decision processes, and therefore involves multiple brain regions. Specifically, correct performance of this task relies on intact prefrontal (Hasselmo, 2005) and medial temporal lobe, particularly hippocampal (Martin and Clark, 2007) neural circuitry, and their functional interactions (Floresco et al., 1997; Muzzio et al., 2009).

**Figure 1:**
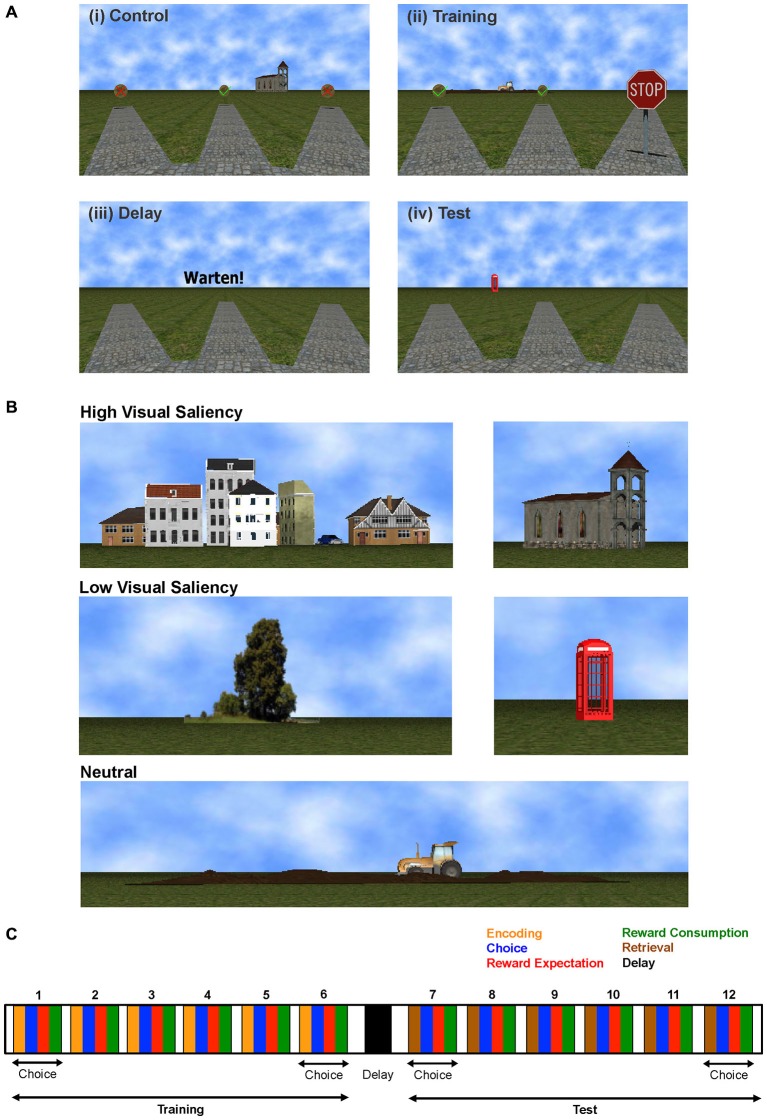
**Radial Arm Maze Task. (A)** Task Stages: (i) Visuo-motor control phase, (ii) Training phase, (iii) Delay phase, and (iv) Test phase for each trial. **(B)** Landmarks of different visual saliency. **(C)** Experimenter defined task stages for one task trial consisting of training and test phases, each comprising six choices where the subject encodes (during training) or retrieves (during test) working memory information, chooses an arm, expects and (if correct) consumes the reward. Time on each task stage is completely self-paced.

Here, we used a virtual reality version of the RAM task (Figure 1), designed in our laboratory (Bähner et al., 2015) to be as compatible as possible to that found in the rodent literature (Brown and Giumetti, 2006; Lapish et al., 2008). We demonstrate that our novel approach reveals valuable insight into region-specific neural processing during task execution, and could help identify the different roles of brain regions in performing one and the same task.

## Materials and Methods

### Task

We used a virtual reality version of the RAM task implemented for use in humans (Figure 1A). Task software was written in C++ using OGRE 3D virtual reality environment (Bähner et al., 2015). During acquisition of fMRI data, subjects used an fMRI-compatible 4-button diamond-shaped fiber optic response pad (Current Designs, Philadelphia, PA, USA) to navigate through a virtual park surrounded by five landmarks to find gold coins hidden at the end of 12 alleys. These landmarks, namely: houses, church, tree, telephone booth and tractor, are shown in Figure 1B. Their visual saliency was determined by asking a group of ten subjects during task design, which landmarks they considered more salient and hence more useful whilst performing the task. The tractor landmark had ambiguous responses regarding its saliency hence we labeled it as “neutral”.

Subjects were informed that the amount of money they could earn depended on their performance. The paradigm was completely self-paced and consisted of three task (training, delay, test) and matched control phases per trial. During the visuomotor control phase, all arms were baited with visible gold coins. Half of the coins were marked with a green check mark and the other half were marked with a red cross (Figure 1A(i)). Subjects were instructed to collect the six gold coins with a green check and avoid the other arms. This phase was followed by a 30 s control delay period. During training phase, half of the arms were blocked (by STOP signs) and subjects were instructed to collect the six accessible gold coins (visible coins with green check marks) and memorize the location of the blocked arms using landmarks that surround the maze (Figure 1A(ii)). After another 30 s delay period (Figure 1A(iii)), the test phase started—here all the arms were accessible and subjects had to find the remaining gold coins in the previously blocked arms (Figure 1A(iv)). In the test phase, gold coins become visible only after the subject reached the end of an arm. For correct choices, a gold coin with a green check mark appeared; a gold coin with a red cross indicated an incorrect choice. After each phase, the number of correct choices was displayed, including the number of gold coins and the money earned during the respective phase; 10 cents per coin were given in the control and training phases, and 20 cents in the test phase.

### Participants

The study was approved by the local ethics committee of the Medical Faculty Mannheim of the University of Heidelberg in accordance with the Declaration of Helsinki. Participants received written and oral instructions of the procedures, and gave informed written consent. Subjects were informed that they could earn up to 9.60 € depending on their performance.

Participants (*N* = 19; 10 males, 9 females; age: mean ± std = 27 ± 4.4) had no history of psychiatric disorder, and had normal or corrected-to-normal vision. They were first instructed and trained outside the scanner; training was repeated if subjects committed more than three errors; usually not more than 1–2 errors were made. During scanning, all subjects completed five trials and the set of baited arms changed for every run. The task design encouraged the use of a spatial, landmark-based strategy; subjects were asked about their employed strategy after the scan. Performance on the task was scored with two types of behavioral errors automatically recorded, namely: *reentries* into arms previously visited during the same task phase, and *revisiting* of arms in the test phase that have been baited during the preceding training phase.

### Data Acquisition and Preprocessing

BOLD-fMRI data were acquired on a 3T Siemens Trio with a 32-channel head coil using parallel imaging (GRAPPA, PAT 2). A gradient-echo echoplanar imaging (EPI) sequence with the following specifications was used: 33 slices, 3 mm slice thickness, 1 mm gap, *TR* = 1.8 s, *TE* = 30 ms, field of view = 192 × 192 mm, flip angle = 73°, in-plane resolution of 3 × 3 mm. Since the task was completely self-paced, the number of recorded volumes was mean ± std: 997 ± 87, range: [875 1170] volumes, depending on the participant’s performance. The datasets underwent typical fMRI preprocessing, including slice time correction, realignment and spatial normalization to a standard EPI template, using SPM8 (Wellcome Department of Imaging Neuroscience, Institute of Neurology, London, UK). The first four volumes of each run were discarded to account for magnetic saturation effects. No spatial smoothing was applied. Data were standardized (i.e., centered and scaled to have zero mean and unit variance), detrended and high pass filtered at 256 s. Head movement parameters were regressed out, and the time series were deconvolved using a modified version of the SPM function (*spm_peb_ppi.m*) described in Gitelman et al. (2003) in order to adjust for the filtering effects of the hemodynamic response function.

Analyses were conducted on regions of interest (ROIs) defined according to the Harvard-Oxford probabilistic mask atlas^1^, thresholded at 50%. Based on previous human fMRI studies (Astur et al., 2005) and findings from the rodent RAM literature (Lapish et al., 2008; Balaguer-Ballester et al., 2011) we selected ROIs postulated to be involved in task execution, namely the anterior (ACC) and posterior (PCC) cingulate cortices, the orbitofrontal cortex (OFC) and the hippocampus (HC). We also defined areas that we anticipated would be less involved in this task, like the Heschl gyrus (Hes) and the insula (Ins), as “control regions” for results comparison, although our approach does not rely on any such specification (i.e., the term “control region” is merely used in a descriptive, not in a methodological sense, while hypothesis testing was solely based on bootstraps and on contrasting different choice groupings among regions, as explained below). The Heschl gyrus contains the human primary auditory cortex, mainly involved in the processing of auditory cues (Warrier et al., 2009) that were not part of this task, whilst for the insula to our knowledge there are no findings that would indicate a prominent role in spatial working memory.

**Figure 2:**
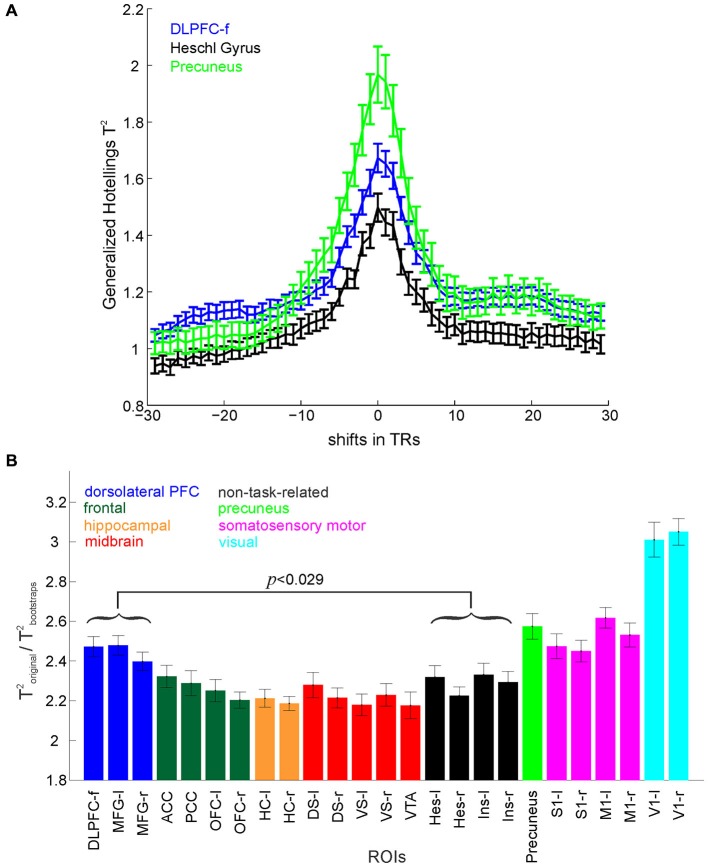
**Multivariate test statistics. (A)** Hotelling’s Generalized T^2^ computed for a set of positive (lags) and negative (leads) shifts of behavioral labels with respect to the time series. The maximum test statistic occurs at zero lag after time-series deconvolution. **(B)** Hotelling’s Generalized T^2^ normalized by the corresponding bootstraps for different ROIs. The DLPFC (represented by 3 masks, MFG left and right, and DLPFC-f) shows significantly higher overall separation of the task stages in comparison to non-task-related areas (*p*-value Bonferroni corrected for multiple comparisons). Error bars = SEM across participants.

Since it is difficult to anatomically define the dorsolateral prefrontal cortex (DLPFC), this ROI was captured by the Harvard-Oxford middle frontal gyrus (MFG) mask, and by an additional empirical mask defined from functional activation, DLPFC-f right (Esslinger et al., 2009) as indicated in Figure 2B. Since the RAM task involves visual stimuli as well as somatosensory and motor demands due to spatial navigation, we also included the primary visual cortex (V1), the primary somatosensory cortex defined as postcentral gyrus (S1), the primary motor cortex defined as precentral gyrus (M1), and the precuneus. For midbrain regions, the ventral tegmental area (VTA) mask was constructed by drawing a region of interest on MRI-based anatomy of the VTA region using an anatomical atlas, and for the dorsal (DS) and ventral (VS) striata the WFU PickAtlas was used (Maldjian et al., 2003, 2004). For most ROIs, the left and right hemispheres were considered separately (Figure 2B). Center coordinates for these ROIs can be found in the Supplementary Material (Table 1). We purposely did not select these ROIs based on maximal voxel activation procedures (Friston, 2007) since we wanted to investigate area-specific information processing rather than the general involvement (activation) of these regions in task execution.

### Data Analysis

**Figure 3:**
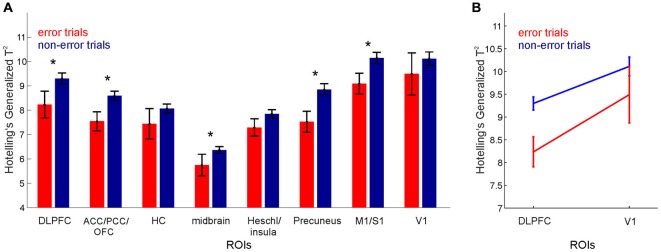
**Error processing.** Hotelling’s Generalized T^2^ for error vs. non-error trials for **(A)** various ROIs. Note that the midbrain ROI result represents the average of the test statistics for midbrain masks (dorsal and ventral striatum, and VTA). **(B)** DLPFC and V1. Error bars, SEM across grouped trials for all participants.

For analyzing the multivariate fMRI BOLD signal time series we employed both *supervised* methods, which required the assignment of a behavioral label to every time point, and an *unsupervised* approach, which derived information from the BOLD data without *a-priori* behavioral class label information. For most of the supervised analyses reported here, the time on task was split into nine cognitively defined task stages, and each time point was assigned one of the following behavioral labels: *choice* (two time points before arm entrance), *reward expectation* (duration of traversing from arm entrance to reward) and *reward consumption* (two time points from reward acquisition), during training and test phases respectively. Navigation at the center of the maze was labeled as *encoding* during the training phase and *retrieval* during the test phase, whilst the delay phase time points were labeled as *delay*, as shown in Figure 1C. Time points corresponding to behavioral errors were excluded from the multivariate analyses, with the exception of the error processing analysis reported in Figure 3. Although not a principle requirement for our approach, the control task was also divided into these task-epochs and for each class, the mean of the control activity per trial was subtracted from the corresponding training, delay and test phase classes. Here this was done to compensate for the strong sensory-motor demands incurred by this task, and to shift the focus more toward the higher cognitive processes of most interest, not to strictly isolate any particular process.

### Multivariate Test Statistics for Quantifying Differences Among Task-Related Activity Patterns

Three multivariate test statistics (as typically employed in MANOVA) were computed separately for each ROI, namely Roy’s greatest characteristic root (GCR; defined as the maximum eigenvalue of ${\hat{\sum }}_{\text{w}}^{-1}{\hat{\sum }}_{\text{b}}$, where ${\hat{\sum }}_{\text{w}}$ and ${\hat{\sum }}_{\text{b}}$ are the properly scaled within- and between-groups covariance matrices, respectively; see, e.g., Krzanowski, 2000; Haase, 2011), Wilk’s lambda defined as det $\left({\hat{\sum }}_{\text{w}})/\det({\hat{\sum }}_{\text{w}}+{\hat{\sum }}_{\text{b}}\right)$, and Hotelling’s Generalized T^2^ defined as trace (${\hat{\sum }}_{\text{w}}^{-1}{\hat{\sum }}_{\text{b}}$). These test statistics provide a measure of the overall discriminability of the nine different task stages (defined above) within the patterns of brain activation in a particular ROI. This represents the amount of information about the different task stages contained within the multivariate voxel patterns. Since time series data violate the independence assumption of conventional parametric statistical testing, significance of classification results was tested non-parametrically against time series (block-permutation) bootstraps. Bootstraps were constructed by shuffling, for each trial, whole blocks of identical consecutive class labels corresponding to a given task stage, hence preserving autocorrelations within the original data. To account for the variable number of voxels in the different ROIs, for every ROI, the test statistics and their corresponding bootstraps were computed 50 times with *n* voxels chosen at random from the voxels within that ROI, where *n* is the total number of voxels within the smallest ROI. The average value was computed as the test statistic for that ROI, and tested for significance across the pooled bootstrap distribution (50 × 1000 bootstrap values). This procedure was repeated for every participant and the average across participants is shown in Figure 2B.

In order to test that the deconvolution procedure (Gitelman et al., 2003) did indeed adjust for the BOLD delay incurred by the hemodynamic response function, Hotelling’s Generalized T^2^ was computed for a series of positive (lags) and negative (leads) shifts of behavioral labels with respect to the deconvolved time series for different ROIs. T^2^ values peaked at a lag of zero (i.e., high values indicate a better class separation, see Figure 2A), confirming that the alignment of task phase labels and the deconvolved BOLD time series was indeed optimal for this lag.

The extent to which regional-specific neural patterns were altered during poor task performance was then assessed by computing Hotelling’s Generalized T^2^ for each trial separately, for every participant and for every ROI. T^2^ values for those trials containing behavioral errors were then grouped across participants and compared with those containing no errors (Figure 3).

### Testing Information Processing Hypotheses Using Different Classification Schemes

To specifically investigate the contribution of different ROIs to various aspects of task processing, different classification schemes were set up based upon a set of *choice* time points (of which there were 12 in total per trial, 6 within each training and test phase, as shown in Figure 1C). To test the responses of brain areas to working memory load, 8 of these 12 choice points were assigned to one of two memory load conditions, namely the first two choices at the start of the training phase (Figure 1C, choices 1 and 2) and the last two choices at the end of the test phase (Figure 1C, choices 11 and 12) into the *low memory load* condition (as the subject at these points in time would only have to retain 2 items in working memory), while the last two choices of the training phase (Figure 1C, choices 5 and 6) and the first two choices of the test phase (Figure 1C, choices 7 and 8) were assigned to the *high memory load* condition (since with either a pro- or a retrospective code at least 4 items would have to be retained). Differences between these two classes across ROIs were quantified through Mahalanobis distances (MD; Krzanowski, 2000), and tested against block permutation bootstraps (as described above).

This *Memory Load* grouping was compared to: (a) *Visual Saliency Grouping*, where choice blocks were grouped into two sets of four arms associated with *high* (houses and church), and *low* (tree and telephone booth) visually salient landmarks (Figure 1B). The tractor landmark had ambiguous responses regarding its saliency and was thus omitted from this analysis; (b) *Random Grouping*, where two groups of four choice blocks chosen randomly without replacement were considered; the random sampling was repeated 10 times, and block permutation bootstraps were also computed for each instance. The average Mahalanobis distance (across the 10 instances) between the two groups was then considered for further comparison to the *Memory Load* and *Visual Saliency* results. Only blocks of *correct* choices were considered for all analyses to avoid confounding the results by behavioral errors.

**Figure 4:**
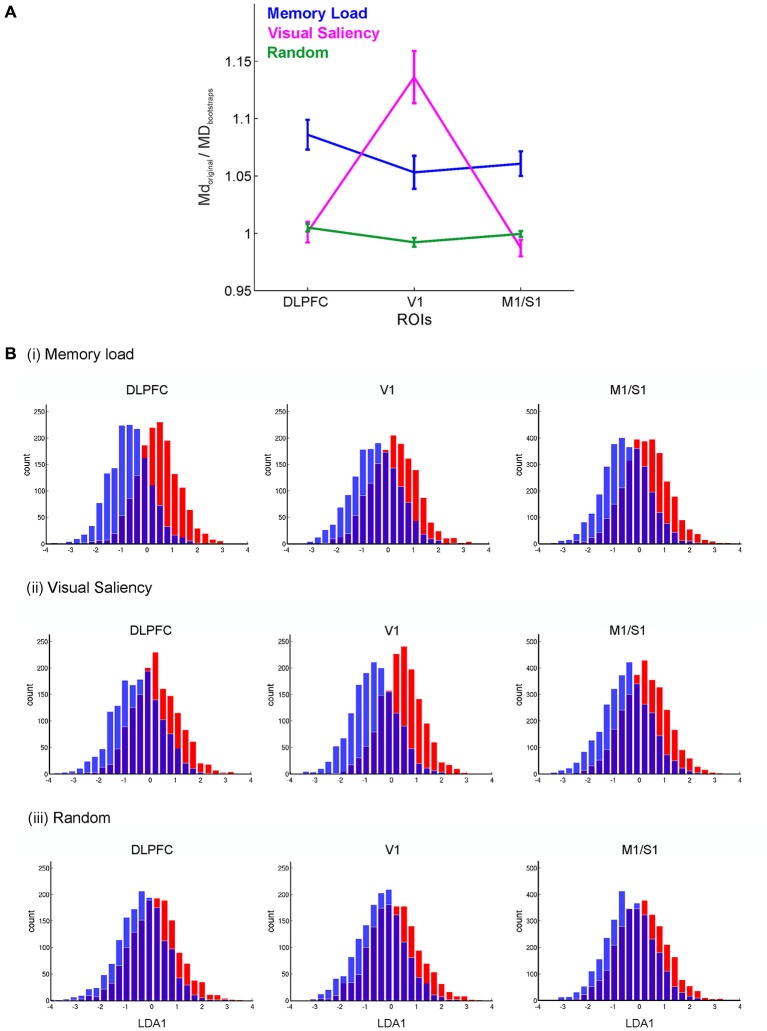
**Decoding of the choice process for different classification schemes. (A)** Mahalanobis distances (MD) normalized by the corresponding block-permutation bootstraps between two sets of decision blocks grouped by *high/low memory load, high/low visual saliency*, and *randomly*, for the DLPFC, V1 and combined motor and somatosensory (M1/S1) regions. Error bars = SEM across participants. **(B)** Distribution of choice time points projected onto the most discriminating direction obtained by Fisher’s LDA. These results show the prominent role of the DLPFC in working memory, and that of V1 in processing visual saliency. Histograms represent *z*-scored projections onto the first Fisher’s LDA direction (LDA1) pooled across participants.

To visualize and confirm regional preferences for certain aspects of task processing (as more formally analyzed through MD), we used regularized Fisher’s linear discriminant analyses (LDA) on choice time points grouped by: (a) Memory Load; (b) Visual Saliency; and (c) Randomly, each comprising two classes (as described above). Fisher’s LDA seeks directions in voxel space along which differences among class means are maximized whilst within-class scatter is minimized (Hastie et al., 2011). Regularized Fisher’s LDA deals with the problem of high dimensional sparse spaces by regularizing (implicitly reducing the degrees of freedom) the estimate ${\hat{\sum }}_{\text{w}}$ of the pooled *within-class* covariance matrix, ${\hat{\sum }}_{\text{w}}=(1-\xi ){\hat{\sum }}_{\text{w}}+\xi \text{diag(}{\hat{\sum }}_{\text{w}}\text{)}$, where ξ is the regularization parameter, empirically set to 0.2 for this dataset. Prior to LDA, the dimension of each ROI was reduced by *k*-medoids (Hastie et al., 2011). This algorithm selected a subset of voxels (*k* = 15 here) that optimally represented (in a prototypical sense) the set of all voxels within that ROI. Distributions of class members projected onto the most discriminating LDA direction (LDA1) were then plotted for visualization. Each participant’s projection onto LDA1 was first *z*-transformed to bring all participant’s projections onto a common space, and then pooled across participants, as shown in Figure 4B.

### Unsupervised Analysis of Cognitive States by Hidden Markov Models

In contrast to the supervised analyses that rely upon experimenter-defined class labels, we also approached the idea of discerning different processing stages from the multivariate BOLD patterns using an unsupervised approach, namely Hidden Markov Models (HMMs; Rabiner, 1989; Bishop, 2007). HMMs automatically dissect the time on task into different underlying states—an approach that is especially suitable for the self-paced nature of this task. A HMM assumes that the observed variables (multivariate voxel patterns) are generated by underlying “hidden” states through which the system progresses, and that have to be estimated from the data. Thus, a HMM *M* = {*A,π,θ*} is defined by a matrix *A* = (*a*_ij_), *a*_ij_ = *P*(s_j_|s_i_), of transition probabilities among predecessor states *s_i_* and successor states *s_j_*, a set θ = {θ_i_} of parameters specifying conditional “emission” densities (here taken to be Gaussian) *f*_θ_(*v*|s_i_) of observation vectors *v* given states *s_i_*, and a vector *π* of initial probabilities π_i_ = *P*(s_0_ = *i*). *A, θ* and *π* are parameters that need to be estimated from the data, together with the unknown states themselves. Based on these, the sequence of states that optimally (with maximum likelihood) describes the data at hand can be determined.

**Figure 5:**
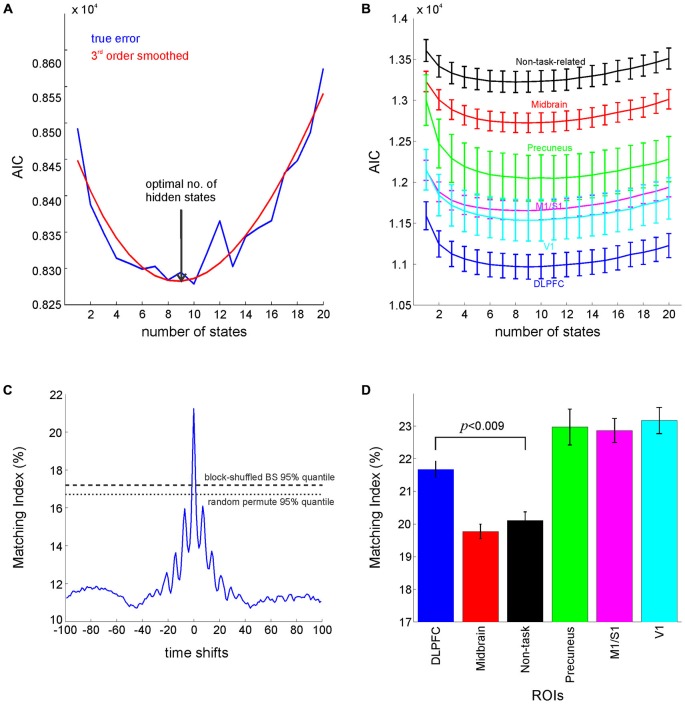
**Hidden Markov Models Analysis. (A)** AIC (raw: blue, smoothed: red) for the DLPFC attains a minimum around nine states. **(B)** The goodness of fit (as ssessed by AIC) for different ROIs. **(C)** Time-shifting bootstraps for testing the significance of the Matching Index between the Viterbi sequence and sequence of behavioral labels, showing significantly higher matching index at zero lag when compared to block or random permutation bootstraps (α = 0.05). **(D)** Matching index for different ROIs (*p*-value Bonferroni corrected for multiple comparisons) computed as percentage of times points where the behavioral sequence matched the Viterbi sequence. Error bars = SEM across participants. Midbrain label refers to the average of results for midbrain masks (dorsal and ventral striatum, and VTA), and non-task-related label refers to the average of results for Heschl gyrus and insula.

HMMs were implemented according to the following procedure:

1. To facilitate HMM building, given the prohibitively high dimensionality of the voxel time series, the *k*-medoids algorithm (Hastie et al., 2011; see above) was first run in order to select a subset of voxels (*k* = 5) which optimally represent the set of all voxels within a ROI. This limits the number of multivariate observations upon which the HMM is built in an attempt to reduce dimensionality by exploiting redundancy.
2. A series of models (with the number of hidden states varying from 1–20) were constructed based on this subset of most representative voxels derived in (a). The states and the model parameters were determined by means of the expectation-maximization algorithm for which an R package for HMMs, *depmixS4*, was used (Visser and Speekenbrink, 2010).
3. The Akaike information criterion (AIC) was computed for each model as a measure of the overall goodness of fit of the model given the data and number of free parameters (Visser and Speekenbrink, 2010). The optimal number of hidden states was selected based on the global minimum of the AIC for different models, smoothed by a third order polynomial, as shown in Figure 5A.
4. For the optimal model, the Viterbi algorithm (Bishop, 2007; Visser and Speekenbrink, 2010) was used to recover the sequence of states that is most likely to have generated the output sequence of observation vectors (a.k.a. the Viterbi sequence) given the model parameters.
5. A Matching Index was defined to capture the correlation between the Viterbi sequence and the experimenter-defined sequence of task labels, computed as follows: (i) Both sequences were converted into a set of binary {0,1} “design” vectors (one for each state or behavioral class), where “1” indicates the presence of a specific state (Viterbi sequence) or behavioral class (experimenter-defined labels), while “0” indicates its absence; (ii) Pearson’s correlation coefficient was computed for each pair of binary sequences, and each Viterbi state was assigned to the behavioral class with which it had the highest correlation; (iii) The behavioral sequence was reconstructed using the newly assigned class-to-state labels and compared to the Viterbi sequence with Matching Index = (Number of Hits/Sequence Length)*100 i.e., expressed as a percentage; and (iv) Significance of the Matching Index was tested non-parametrically against cycle shifting, block- and random permutation bootstraps, as shown in Figure 5C.

## Results

### Assessing the Involvement of Different Brain Regions in RAM Task Performance

We investigated information processing in different brain areas during the performance of the RAM task by means of a variety of supervised multivariate statistical and time series methods employed on the multivariate BOLD data. The cognitive task epochs (classes) included time points associated with *choice*, *reward expectation* and *reward consumption* for the training and test phases respectively, *delay*, *encoding* (training) and *retrieval* (test). We used common multivariate test statistics as defined within multivariate general linear models (Haase, 2011), which measure the overall discriminability of the different task stages within the BOLD signal pattern. In order to account for potential ROI-specific differences in the (auto-) correlative properties of the multivariate BOLD time series, the raw test statistics were normalized by the same quantities computed from block permutation bootstraps, which preserve the original auto-correlations (see Section Materials and Methods). Figure 2B shows group results (across all participants) for Hotelling’s Generalized T^2^ normalized by the corresponding bootstraps (Roy’s GCR and Wilk’s Λ gave similar results).

When raw T^2^ values averaged across participants were compared non-parametrically to their respective bootstrap distributions, significance (*p* < 0.05) was achieved for all ROIs, indicating that in fact all investigated brain areas contain a significant amount of information about the task environment as a whole (i.e., not distinguishing between sensory, motor, or cognitive aspects of the task). Furthermore, for all participants, test statistics achieved significance (*p* < 0.05) individually if compared non-parametrically to their respective bootstrap distributions, demonstrating that significance of task-related information could be established at the single-subject level. Although, in general, all included ROIs contained a significant amount of information about task aspects, comparing different ROIs directly through repeated measures ANOVA revealed a significant main effect across regions (when considering the eight grouped sets of ROIs, as shown in Figure 2B: *F*_(7,126)_ = 68.92, *p* < 10^–6^), which indicates that there are still regional variations in task information. The highest discrimination of task stages was achieved in the V1, followed by the precuneus, somatosensory-motor (S1 and M1) and prefrontal regions, whilst the lowest was found in control (Heschl gyrus and insula) and in midbrain regions. Furthermore, pairwise comparisons (paired *t-tests*, Bonferroni corrected for multiple comparisons) revealed that overall task stages were better separated in the DLPFC (represented by three masks, MFG left and right, and DLPFC-f) than in non-task-related regions (*t*_(18)_ = 3.90, *p* < 0.029). Moreover, Figure 2B suggests that from all the regions postulated to be involved in this task, such as the ACC, OFC and the HC, the DLPFC contains the most information on task structure.

Hotelling’s Generalized T^2^, computed separately for every trial, was then used to investigate how information processing changed during poor task performance. Across all 19 participants (giving a total of 19 × 5 task trials) there were 10 error trials and 85 non-error trials. A 2-way ANOVA with *region* (8 ROIs grouped as shown in Figure 3A) and *error grouping* (error vs. non-error) as factors, revealed a significant main effect for region (*F*_(7,744)_ = 38.88, *p* < 10^−6^), and for error grouping (*F*_(1,744)_ = 14.13, *p* < 0.0002), hence demonstrating an overall reduction in T^2^ values as a function of behavioral errors across the regions considered (Figure 3A). This reduction in T^2^ values during error trials is shown in all regions except in HC, the Heschl gyrus and insula (postulated to be non-task-related, control regions for this task), and in V1 despite its high discriminability between the task stages. Moreover, pairwise comparisons (paired *t*-tests, Bonferroni corrected for multiple comparisons) showed that T^2^ values for error trials were significantly lower than those for non-error trials for the DLPFC (*t*_(18)_ = 2.45, *p* < 0.02), but not for V1 (*t*_(18)_ = 0.98, *p* < 0.33), as shown in Figure 3B. This lower discriminability of cognitive stages is consistent with impaired area-specific neural processing during poor performance in those regions which are highly task-relevant, similar to that observed in rodents on the same kind of task using multiple single-unit recordings (Lapish et al., 2008).

The multivariate test statistics such as Hotelling’s Generalized T^2^ used above indicate the regions that contain a significant amount of information about the experimenter-defined task stages, but they do not tell us which stages specifically, or which precise aspects of these task stages are most discriminative for any given region. For instance, the stages defined above differ in both cognitive and visual characteristics, and hence different brain regions could have selectively responded to these characteristics. To explore this further, the time points associated with correct *choices* were grouped according to various functional hypotheses. Specifically, time points associated with *choices* were grouped into either two classes of: (a) low vs. high *working memory load*; (b) low vs. high *visual saliency* of the landmarks associated with the arms; or (c) *random* assignments (see Section Materials and Methods). Figure 4A shows the MD (Krzanowski, 2000) between sets of BOLD vectors associated with pairs of such defined class contrasts, normalized by the corresponding bootstraps, for the DLPFC (averaged across the MFG left and right, and DLPFC-f masks), V1, and primary somatosensory and motor areas (labeled M1/S1 grouped across left and right regions) for groupings according to the *working memory load*, the *visual saliency* of the landmarks associated with the chosen arms, and for *random* assignments of choice arms to groups (see Section Materials and Methods). A repeated two-way ANOVA with region (DLPFC, V1 and M1/S1 areas) and grouping (*Memory load*, *Visual saliency, Random*) as factors revealed a significant region x grouping interaction (*F*_(4,72)_ = 22.37, *p* < 10^–6^), as well as a significant main effect for region (*F*_(1.91,34.3)_ = 9.29, *p* < 0.001), and for grouping (*F*_(2,36)_ = 9.47, *p* < 0.0004); degrees of freedom were Greenhouse-Geisser corrected where appropriate. Moreover, pairwise comparisons showed that the separation of low/high visual saliency choices was significantly higher in V1 in comparison to the DLPFC (*t*_(18)_ = 4.59, *p* < 0.0007), and significantly higher than that obtained for random grouping (*t*_(18)_ = 4.67, *p* < 0.0003). In contrast, for the DLPFC, the separation of low/high memory load choices was significantly higher than that obtained in V1 (*t*_(18)_ = 3.36, *p* < 0.009), whilst that for visual saliency choices did not significantly differ from random grouping (*t*_(18)_ = 0.27, *p* > 0.5). Meanwhile somatosensory-motor regions, which also achieved high overall discrimination among task stages (Figure 2B), did not seem to be either particularly sensitive to visual saliency or to memory load choices (Figure 4A). Thus, while all regions contained *some* information about task events (with V1 even surpassing DLPFC in overall task stage discrimination, as shown in Figure 2B), this result reveals the specific and separable contribution of both regions to task performance.

Finally, to add visual intuition to the class discrimination revealed by the Mahalanobis statistics, Fisher’s LDA (Hastie et al., 2011) was employed to seek out directions in the ROI voxel space along which the defined classes were optimally separable. Figure 4B shows the distribution of choice time points projected onto the most discriminant direction obtained by Fisher’s LDA, after first reducing the dimensionality in voxel space through *k*-medoids (see Section Materials and Methods). These histograms visually confirm the Mahalanobis distance results of better DLPFC discrimination among memory loads, and better V1 discrimination among visual saliency conditions.

### Unsupervised Identification of the Cognitive Processing Stages

In the analyses above, we assigned time points to task labels based on our own assumptions about cognitive processing steps or cognitively separable events (i.e., *supervised* classification). Alternatively, in an *unsupervised* approach, one may look for “natural groupings” of time steps within the multivariate BOLD time series that may reflect task processing. This may be particularly revealing in the present case because of the self-paced nature of the task, which implies that the exact timing of some cognitive acts (such as *choice* and *reward expectation*) is not rigidly constrained experimentally (although the results in Figure 2A, using cycle-shifting bootstraps, may partly support our choice of temporal boundaries for the different task events). Therefore, we used HMMs as a complementary unsupervised approach for identifying underlying information processing steps (hidden states) and their sequential timing without relying on experimentally-defined class labels.

A series of models with varying numbers of hidden states were built for the different ROIs, and the AIC (based on the model likelihood) was computed as a measure of model performance taking into account its number of free parameters (growing quadratically with the number of states). The AIC achieved a minimum at nine states as shown in Figure 5A, which happens to agree with the number of experimenter-defined behavioral labels used in the supervised methods. Regional differences in AIC curves (averaged across participants and across grouped regions; Figure 5B) also follow those obtained with multivariate test statistics, with the best goodness of fit achieved for models in DLPFC and V1, and the worst for non-task-related regions. To assess to which degree the nine classes revealed by the HMM analysis actually agreed with the supervised class definitions, we derived a matching index that captured the congruence between the HMM-derived (Viterbi) state sequence and the experimenter-defined sequence of events, i.e., sequence of behavioral class labels, (see Section Materials and Methods). Bootstrap testing (using cycle-shifting block permutations, and random shuffling bootstraps at α=0.05) revealed that the level of agreement between these two sequences was significantly higher than chance (Figure 5C). Moreover, repeated measures ANOVA computed on the six grouped regions (shown in Figure 5D) revealed a significant main effect across regions (*F*_(5,90)_ = 18.26, *p* < 10^–6^), whilst pairwise comparisons (paired *t*-test, Bonferroni corrected for multiple comparisons) revealed significantly higher matching indices for the DLPFC in comparison to non-task-related regions (*t*_(18)_ = 4.2, *p* < 0.009). Thus, regional variations in the matching index mirror those obtained with the supervised procedures, with the highest matching obtained for prefrontal regions, V1, M1/S1 and the precuneus, and the lowest for non-task related and midbrain regions (Figure 5D).

## Discussion

### Area-Specific Information Processing in a Translational RAM Task

In this study we present a novel methodological approach for discerning area-specific contributions to information processing in a complex task, based on a set of multivariate statistical methods and time-series bootstraps. We apply them to data from healthy adults during a spatial multiple-item working memory (RAM) task, and use them to: (i) investigate the discrimination of different cognitive stages in various ROIs; and (ii) derive the specific functional contribution of different ROIs during task execution. Our results demonstrate that anatomical regions postulated to be involved during RAM, such as the DLPFC, precuneus, somatosensory motor and primary visual areas, show clear discrimination of the task stages, which distinguishes them from control areas that are not so relevant to this task, mainly the Heschl gyrus and insula.

It is emphasized, however, that defining a set of regions as “control, non-task-related regions” in the present study was not crucial for hypothesis testing, but was merely included to distinguish between regions expected to be involved in the task, based on previous evidence from human (Astur et al., 2005) and rodent work (Floresco et al., 1997; Lapish et al., 2008; Balaguer-Ballester et al., 2011), vs. those which were not expected to play a major role. The significant and generally high test statistics found even for control regions (Figure 2) may reflect that all brain regions are highly interconnected, and that these presumably non-task-related areas may be receiving inputs from higher executive areas such as the prefronal cortex. Alternatively, it may also be explained by more general task demands or features such as changes in attention or alertness, or general metabolic requirements that may affect the whole brain and that vary throughout the task.

Task-epoch discriminability, as formally quantified by multivariate test statistics (Figure 2B), was further found to be lower during poor task performance (Figure 3), in line with in-vivo multiple single-unit recordings from rodents subjected to the very same kind of RAM task and statistical analysis (Lapish et al., 2008). This is notable given the coarse temporal and—compared to single unit recordings—spatial nature of fMRI BOLD data, which, one would assume, would obscure any finer-grained computational changes.

The segregation of the time-on-task into different discernible cognitive stages was further corroborated by a completely unsupervised algorithm, namely HMMs using the AIC for model selection. HMMs tended to split the time on task in a similar manner as defined by the experimenter, but only in task-related, specifically prefrontal, and not in non-task-related, control regions. This is remarkable, and advertises HMMs as a more general tool for gaining insight into cognitive processing from fMRI data in situations where no or only weak assumptions about the cognitive stages involved are preferred.

Furthermore, we show that arranging time points into classes according to different functional hypotheses, in combination with multivariate classifiers and time series bootstraps, provides a way for disentangling different information processing aspects assessed on the same task, and regional-specific contributions to them. Specifically, grouping choice time points according to different schemata revealed that the DLPFC differentiates significantly stronger than V1 between working memory load conditions, while V1 differentiates more strongly among groups defined by visual saliency. While, at first glance, this result may not be too surprising as the functional specializations of the DLPFC and visual cortex seem well established (e.g., (D’Esposito and Postle, 1999; Postle et al., 1999; Rypma and D’Esposito, 1999), it nevertheless demonstrates the power of the multivariate statistical techniques employed here since the differential involvement of the two areas was demonstrated *based on the very same BOLD time series*, obtained during the *same task* that activated both areas, in contrast to opposing them by task design. Furthermore, it should be noted that there are also findings which suggest that the PFC is also sensitive to visual saliency and object information (Rainer et al., 1998) while—*vice versa—*V1 neurons have also been demonstrated to carry working memory information (Supèr et al., 2001).

The significant involvement of the precuneus in the RAM task is also in line with recent evidence in the literature, where this posterior region of the medial parietal cortex has been implicated in a wide range of higher-order cognitive functions (for review see; Cavanna and Trimble, 2006), and has been demonstrated to be a central hub region in the human connectome, heavily connected with several other cortical and subcortical regions, including the DLPFC (van den Heuvel and Sporns, 2011). In human literature, several fMRI and PET studies of visuo-spatial imagery have shown significant activation of the precuneus, both during the execution (Kawashima et al., 1995) and preparation (Astafiev et al., 2003) of spatially guided behaviors, and for directing attention in space during the execution of goal-directed movements. The study by Ghaem et al. (1997) suggests that both visuo-spatial imagery and retrieval processes during mental navigation could be related to precuneus activation.

The dorsal and ventral subregions of the striatum are thought to be differentially involved in spatial working memory (De Leonibus et al., 2005). The VS forms part of the reward system and is thus more involved in reward processing, whilst the DS contributes directly to decision-making aspects of the task (Balleine et al., 2007). However, we did not observe prominent significant differences between these two subregions or between these regions and the VTA (Figure 2), for both our supervised and unsupervised methods.

The significantly highest task-stage discrimination in V1 (in comparison to the other ROIs, cf. Figure 2B) can be attributed to the visual variations of the task stages as one navigates around the maze. Since V1 contains the largest and most detailed general-purpose representations of the visual field (Bullier, 2001), it naturally picks up on these differences. The much weaker differentiation between low and high memory load conditions in V1 compared to the DLPFC, despite an overall better discrimination among task periods, speaks against a prominent role of visual areas in the active maintenance of training phase information. Meanwhile, the high discrimination within somatosensory motor areas (M1/S1) can most likely be attributed to the different button-press requirements of the task stages—for example, the delay phase does not require a button press, in contrast to the other stages. Our results (cf. Figures 2B, 5D) may at first sight suggest that the primary sensory cortex is to some degree also involved in the transient storage of working memory information, similar to primate single unit findings reported by Harris et al. (2001, 2002) during a tactile working memory task. However, our pairwise results suggest that its involvement in working memory is significantly weaker than that of the DLPFC (Figure 4).

### The Benefits of Employing Multivariate Approaches on fMRI BOLD Data

Our results demonstrate that different cognitive stages during the performance of the RAM task can be identified and separated from the BOLD time series using both supervised and unsupervised multivariate methods. These techniques allow the extraction of information represented in distributed patterns of BOLD signals, and may thus provide more fine-grained information about task-related neural representations and processing, beyond confirming that an area plays a role in the task (Hampton and O’Doherty, 2007; Haynes et al., 2007; Soon et al., 2008; Kriegeskorte, 2011). Furthermore, we show that they can be used to assess regional-specific contributions and/or variations in information processing during one and the same task.

In massively univariate fMRI analyses, often a statistical threshold is used to decide whether a brain area is activated by the task. However, failure of a given brain region to survive such statistical thresholding does not necessarily imply that it is not involved in the task. If a computational process does not result in a change of the overall activity level in a given brain area (Logothetis, 2002), it may not be detected by these analyses (O’Doherty et al., 2007). For instance, if neural populations within a ROI exhibited opposing firing rate encoding schemes (e.g., increase/decrease of firing rates with increasing/decreasing stimulus value), the main effect may be cancelled out (Kahnt et al., 2011b). Although multivariate techniques still function within the spatio-temporal limitations inherent in fMRI BOLD data, they go a step beyond and allow the extraction of information represented in distributed patterns of neural activity, even if these patterns are not accompanied by an overall change in mean firing rates.

To date, most of the applications of multivariate classifier techniques in fMRI research (Haynes and Rees, 2005, 2006; Norman et al., 2006; Soon et al., 2008; Bode and Haynes, 2009; Kahnt et al., 2011a) have been based on tasks purposely designed with different behavioral events well separated in time. Whilst such designs are certainly advantageous for clearly discriminating and predicting different mental states and/or task stages from BOLD activity patterns, they do not easily allow the processing steps to be teased apart during continuously evolving, temporally extended tasks like the RAM. In this work we purposely used a task that is completely self-paced, and hence the exact timing of some cognitive acts was not experimentally constrained. Moreover, the different instances from the classes to be discriminated were not widely separated in time. Our results demonstrate that despite the considerably poor temporal resolution of fMRI BOLD data, task stages following closely in time can be statistically discriminated, hence yielding useful information about different temporally consecutive processing steps and their association with different brain areas.

We suggest that employing *unsupervised* multivariate methods can provide further insight into the underlying neuronal processing. Supervised methods are hypothesis-driven, typically based on task-designed segmentation; hence they may miss out on some aspects of the true temporal-cognitive segregation of the task flow as employed by the underlying cortical information processing system. On the other hand, unsupervised methods such as HMMs simply assume that the brain moves through discrete sets of task-stage-specific activity patterns and cognitive stages, but do not impose restrictions on assessing how it goes about in executing the task, and with which specific timings it does so. Other recent work has also demonstrated the potential of employing multivariate pattern analyses and HMMs on fMRI data to unravel the mental states involved in problem-solving during memory games (Anderson et al., 2012) and in novel mathematical problem-solving (Anderson et al., 2014).

We think that this analysis framework is also promising for clinical studies, to assess how multivariate activity patterns, measured by fMRI, are altered in psychiatric disorders such as schizophrenia.

## Funding

This work was funded through a grant from the German Ministry of Education and Research (BMBF, 01GQ1003B) and from the German Science Foundation to DD (DFG, Du 354/6–1, Du 354/7–2).

## Conflict of Interest Statement

AM-L received consultancy fees from Astra Zeneca, Elsevier, F. Hoffmann-La Roche, Gerson Lehrman Group, Lundbeck, Outcome Europe Sárl, Outcome Sciences, Roche Pharma, Servier International, Thieme Verlag; travel expenses from Abbott, Astra Zeneca, Aula Médica Congresos, BASF, Groupo Ferrer International, Janssen-Cilag, Lilly Deutschland, LVR Klinikum Düsseldorf, Servier Deutschland, Otsuka Pharmaceuticals; and grants from Hans-Jörg Weitbrecht Award, ECNP Neuropsychopharmacology Award, Prix ROGER DE SPOELBERCH. The other authors declare that the research was conducted in the absence of any commercial or financial relationships that could be construed as a potential conflict of interest.

## Footnotes

^1^http://fsl.fmrib.ox.ac.uk/fsl/fslwiki/Atlases/
