# Supplementary material for: A statistical approach for segregating cognitive task stages from multivariate fMRI BOLD time series
Source: Front Hum Neurosci. 2015 Oct 7;9:537. doi: 10.3389/fnhum.2015.00537 (PMC4617410; doi:10.3389/fnhum.2015.00537)
Supplement: Supplementary file 1 [file Table_1.docx]

***Supplementary Material***

**A statistical approach for segregating cognitive task stages from multivariate fMRI BOLD time series**

**Charmaine Demanuele^*^, Florian Bähner, Michael M. Plichta, Peter Kirsch, Heike Tost, Andreas Meyer-Lindenberg, Daniel Durstewitz**

**Correspondence:** Charmaine Demanuele: charmd@nmr.mgh.harvard.edu

**Supplementary Table 1. ROI coordinates in MNI space.** The masks were normalized to a standard EPI template in SPM8 to be in the same space and voxel size as the subjects' data. Coordinates of the center of the masks were derived by overlaying the masks onto a standard anatomical template image (ch2better.nii) in MRIcron.

| **ROI** | **x** | **y** | **z** |
| --- | --- | --- | --- |
| DLPFC-f right | 42 | 36 | 30 |
| Middle frontal gyrus -l | -39 | 24 | 44 |
| Middle frontal gyrus -r | 40 | 22 | 45 |
| Anterior cingulate cortex | 0 | 21 | 26 |
| Posterior cingulate cortex | 0 | -34 | 33 |
| Orbito frontal cortex -l | -26 | 23 | -18 |
| Orbito frontal cortex -r | 26 | 21 | -18 |
| Hippocampus -l | -24 | -12 | -20 |
| Hippocampus -r | 25 | -16 | -18 |
| Dorsal striatum -l | -10 | 10 | 12 |
| Dorsal striatum -r | 11 | 10 | 13 |
| Ventral striatum -l | -8 | 14 | -1 |
| Ventral striatum -r | 8 | 15 | -1 |
| Ventral tegmental area | -2 | -12 | -13 |
| Heschl gyrus-l | -48 | -17 | 6 |
| Heschl gyrus -r | 48 | -16 | 6 |
| Insula -l | -40 | 2 | -3 |
| Insula -r | 42 | 1 | -3 |
| Precuneus | -2 | -64 | 36 |
| Postcentral gyrus (S1) -l | -60 | -12 | 32 |
| Postcentral gyrus (S1) -r | 60 | -12 | 34 |
| Precentral gyrus (M1) -l | -52 | 0 | 42 |
| Precentral gyrus (M1) -r | 53 | -4 | 49 |
| Primary visual cortex (V1) -l | -12 | -96 | -8 |
| Primary visual cortex (V1) -r | 12 | -96 | -10 |
